# An exploration of the nomological network of trypophobia

**DOI:** 10.1371/journal.pone.0257409

**Published:** 2021-09-14

**Authors:** Eric Mayor, Andrea Meyer, Alessandro Miani, Roselind Lieb

**Affiliations:** 1 Division of Clinical Psychology and Epidemiology, Department of Psychology, University of Basel, Basel, Switzerland; 2 Institute of Work and Organizational Psychology, University of Neuchâtel, Neuchâtel, Switzerland; Medical University of Vienna, AUSTRIA

## Abstract

**Background:**

Trypophobia is characterised by an aversion to or even revulsion for patterns of holes or visual stimuli featuring such patterns. Past research has shown that trypophobic stimuli trigger emotional and physiological reactions, but relatively little is known about the antecedents, prodromes, or simply covariates of trypophobia.

**Aim:**

The goals of this study were (a) to draw the contours of the nomological network of trypophobia by assessing the associations of symptoms of trypophobia with several constructs that were deemed relevant from past research on anxiety disorders and specific phobias, (b) to compare such associations with those found for symptoms of spider phobia and blood and injection phobia (alternative dependent variables), and (c) to investigate the main effect of gender on symptoms of trypophobia and replicate the association of gender with symptoms of spider phobia and blood and injection phobia (higher scores for women).

**Methods:**

Participants (*N* = 1,134, 53% men) in this cross-sectional study completed an online questionnaire assessing the constructs of interest.

**Results:**

Most assessed constructs typically associated with anxiety disorders (neuroticism, conscientiousness, anxiety sensitivity, trait anxiety, disgust sensitivity, and disgust propensity) were also associated with trypophobia in the predicted direction. All of these constructs were also associated with spider phobia and blood and injection phobia. Behavioral inhibition was negatively associated with trypophobia and spider phobia—contrary to what was expected, but positively with blood and injection phobia. We found no gender difference in trypophobia, whereas women scored higher on spider phobia and blood and injection phobia.

**Discussion:**

Although some differences were observed, the nomological network of trypophobia was largely similar to that of spider phobia and blood and injection phobia. Further studies are needed to clarify similarities and dissimilarities between trypophobia and specific phobia.

## Introduction

Anxiety disorders have been shown to constitute one of the largest groups of mental disorders. Their estimated 12-month (lifetime) prevalence was reported to be 22.7% (33.3%) among women and 13% (22%) among men in the United States [1] and in the range of 5.6% to 19% (13.6% to 28%) worldwide [2]. In particular, specific phobias are a serious health concern in the population that can start during childhood/adolescence: Research has shown specific phobias to be highly represented among these disorders and to have one of the earliest ages of onset [3]. In the United States, their 12-month (lifetime) prevalence was reported to be 12% (16.1%) in women and 5.5% (9%) in men [1]. In Europe, the reported range of 12-month prevalence of specific phobia was 3.1% to 11% [4]. According to the fifth edition of the *Diagnostic and Statistical Manual of Mental Disorders* (*DSM-5*), specific phobias entail “fear or anxiety [that] is circumscribed to the presence of a particular situation or object” [5, p. 198]. Such objects can be, for instance, animals, the natural environment, or blood, injection, and injury, and situations include airplanes and elevators, among others.

Trypophobia has been defined as an aversion to or even revulsion for patterns of holes or visual stimuli featuring such patterns [6] leading to physiological reactions (e.g., cardiovascular [7,8]; haemodynamic [7]; electrodermal changes [8]; pupil constriction [9]; late positive potential amplitude [10]; early posterior negativity [11]) and subjective reactions (disgust more than fear, skin-crawling sensation [6,8,9]) to confrontation with these stimuli. Studies have shown that whereas non-trypophobic individuals presented aversion only to disease-relevant trypophobic stimuli, trypophobic individuals did so to both disease-relevant and disease-irrelevant stimuli [6]. Estimates of the percentage of individuals feeling disturbed by trypophobic images could be 14% or higher [12]. Trypophobia has not yet qualified as a specific phobia according to the criteria of the *DSM-5*; or at best be classified under the unspecific category-specific phobia, Other Type.

Much of the literature on trypophobia has been dedicated to the (neuro)physiological reactions to trypophobic stimuli (e.g., [8,11]) and the characteristics of trypophobic images linked with aversion (e.g., [13]). The nomological network of trypophobia has to date been largely unexplored. Indeed, research has only sporadically examined potential antecedents, prodromes, or simply covariates of trypophobia. Researchers have found that 20% of respondents recruited from a trypophobia support group had one or more specific phobias, 19% had been diagnosed with major depression, 17% with generalized anxiety disorder, but only 2% with obsessive compulsive disorder [14]. Most individuals in this support group reported suffering from mild to severe psychological distress and impairment [14], which is higher than what was found in individuals with specific phobias [15]. Survey-based studies usually used the Trypophobia Questionnaire (TQ) [16] to measure symptoms of trypophobia. A study found a weak relationship between trait anxiety and such symptoms—but only in a cohort of trypophobic individuals; furthermore, the study found no difference in trait anxiety between participants from a trypophobia support group and those from a university group [16]. Generalized anxiety was not found to be correlated with symptoms of trypophobia [13]. Positive relationships were reported between symptoms of trypophobia and, notably, disgust sensitivity [17] and pathogen disgust [6].

## The current study

Research on trypophobia has surprisingly been conducted mostly without considering the literature on specific phobia, despite similarities in theories of the origins of trypophobia and specific phobia. Indeed, several theories on the etiology of both trypophobia and specific phobia have emphasized notions akin to *preparedness*. Preparedness refers to the idea that stimuli reminiscent of evolutionarily relevant threats are more commonly feared than other stimuli [18].

With regard to specific phobia, the preparedness framework was proposed in the 1970s [19] to account for the inability of the then-dominant behaviorist paradigm (e.g., [20,21]) to explain why some classes of stimuli were considered threatening by many individuals before ever being exposed to them, and the difficulty of extinguishing the then-assumed solely ‘conditioned’ response to these classes of stimuli [18]. In a similar vein, non-associative theory has explained the existence of anxious reactions to some stimuli from the first encounter as a fear response that at some point enhanced the odds of survival [22]. Building upon the preparedness framework, fear module theory suggested the existence of a selective and automatic neural circuitry “sensitive to stimuli that have been correlated with threatening encounters in the evolutionary past” [22, p. 485]. This module has further been deemed impervious to conscious influence [23].

With regard to trypophobia, some researchers suggested that trypophobic stimuli might evoke patterns related to dangerous animals in the evolutionary past, such as venomous organisms, which would trigger an aversion response [12]. This explanation was discarded in a study with preschoolers who performed an implicit association test [24]. The second evolutionary explanation was related to the behavioral immune system: Pathogen cues and associated stimuli elicit avoidance due the emotional responses triggered by them (disgust, fear [25]). Individuals suffering from trypophobia might produce overgeneralized disease avoidance responses to trypophobic stimuli that have only a slight resemblance to disease-relevant cues [6]. There has been some support for this explanation, as pathogen disgust significantly predicted aversion to trypophobic stimuli [6]. Another explanation for trypophobia was that discomfort could stem from the repeating patterns themselves (characterized by excess energy at low and midrange spatial frequencies), which could deviate too much from natural images (e.g., [16]). Some studies have found support for this explanation [16,26].

Our study contributes in three main ways to the literature. First, we sought to assess whether several constructs that were deemed relevant from past research on anxiety disorders and specific phobias are associated with trypophobia (Aim A). Indeed, examining variables linked with specific phobias was a first step in discovering the nomological net of trypophobia and whether it was similar to that of specific phobia. One particular reason we considered this issue worth investigating was that whether trypophobia could be grouped under the diagnostic category of a specific phobia has to date not been established, as the argument that disgust reactions to stimuli predominate over fear in trypophobia [9,24] was far from convincing: Notably, a similar pattern has been found in blood and injection phobia [27,28].

Second, we sought to examine whether these associations were also found in spider phobia and blood and injection phobia (Aim B). These two subtypes of specific phobias were chosen because they have frequently been compared in the literature on account of the distinctiveness of their physiological (e.g., [29,30]) and emotional (e.g., [31–33]) features.

Past research has shown women to be more susceptible to anxiety disorders than men, including specific phobias [1], but the validation studies of the instrument used to assess trypophobia did not include tests of gender differences in trypophobia symptoms [8,16]. A third contribution of the study is thus that we sought to determine the impact of gender on trypophobia (Aim C). We also investigated whether such gender effects could be replicated with regard to our other dependent variables (DVs).

We now present the associations in the literature of the constructs we have selected as well as our hypotheses.

### Behavioral inhibition

Behavioral inhibition (the “tendency to withdraw from novel situations” [34, p. 133]) was shown to constitute a generic vulnerability to anxiety and anxiety disorders [35]. Behavioral inhibition (either current or retrospective) was found to be positively related to specific phobias [36–38]. This variable has also been found to be related to disgust sensitivity, which has been associated with specific phobia and emotional reactions to phobic stimuli [39,40]. We hypothesized behavioral inhibition to be positively related to symptoms of trypophobia (H1A), spider phobia (H1B), and blood and injection phobia (H1C).

### Personality

A meta-analysis showed that individuals presenting with specific phobias had higher neuroticism and lower conscientiousness than those not presenting with these disorders [41]. One study failed to establish a relationship between neuroticism and trypophobia [6]. These negative results have not been replicated since. We hypothesised neuroticism to be positively related to symptoms of trypophobia (H2A), spider phobia (H2B), and blood and injection phobia (H2C) and conscientiousness to be negatively related to these DVs (H3A, H3B, and H3C, respectively).

Research has to date been inconclusive with regard to the association of specific phobia with extraversion, agreeableness, and openness to experience: These dimensions of personality reportedly have negative, positive, or non-significant associations with specific phobia in similar proportions, leading to non-significant bivariate associations in a meta-analysis [41]. Here, we analysed the role of these dimensions exploratively to see if they were similarly associated with each of our DVs.

### Anxiety sensitivity

Anxiety sensitivity (the interpretation of anxiety manifestations as dangerous and fear thereof) has been proposed as an explanation for the development and maintenance of social phobia and other anxiety disorders (see [42]). Studies have shown that individuals presenting with anxiety disorders—including specific phobia—had higher anxiety sensitivity scores than non-clinically anxious individuals (e.g., [43]). Another meta-analysis further showed that the level of anxiety sensitivity in specific phobia was not different from that in other anxiety disorders [44]. To our knowledge, no study has examined the relationship of anxiety sensitivity to trypophobia. We hypothesized anxiety sensitivity to be positively related to symptoms of trypophobia (H4A), spider phobia (H4B), and blood and injection phobia (H4C).

### Trait anxiety

Research has shown trait anxiety (the dispositional tendency to be fearful, worried, and/or anxious) to be related to spider phobia and blood and injection phobia (albeit not controlling for disgust propensity) and obsessive-compulsive disorder [45]. Trait anxiety and trypophobia have been found to be related among trypophobic participants but not in other participants [16]. We hypothesized trait anxiety to be positively related to symptoms of trypophobia, (H5A), spider phobia (H5B), and blood and injection phobia (H5C).

### Disgust

Disgust propensity (the propensity to react with disgust) and disgust sensitivity (anxious apprehension concerning experiencing disgust) have been reported to be associated with anxiety disorders generally and with subtypes of specific phobias—with some differences between subtypes [32,33,45]. Disgust sensitivity was found to be related to trypophobia [6,17]. We hypothesized disgust sensitivity to be positively related to trypophobia (H6A), spider phobia (H6B), and blood and injection phobia (H6C). We also hypothesized disgust propensity to be positively related to these variables (H7A, H7B, and H7C, respectively).

### Gender

Previous studies on specific phobias have shown that women are more likely to present with these disorders (e.g., [1]). To further assess the similarity of covariates of trypophobia and specific phobias, we hypothesized women would score higher on trypophobia symptoms (H8A), and we expected to replicate existing findings on gender differences in symptoms of spider phobia (H8B) and blood and injection phobia (H8C).

## Methods

### Participants

Participants (*N* = 1,134, 53% men) were Mechanical Turk workers (MTurkers) with a task acceptance rate of 95% or higher who resided in the United States and successfully passed attention checks. Using an a priori power analysis in G*Power, we obtained the following results for partial correlation (see Data analysis below). Given alpha = .05 and beta = .2, that is, power = .8, for a large effect size (*f*^2^ = .35), *N* = 25 was required; for a medium effect size (*f*^2^ = .15), *N* = 55 was required; and for a small effect size (*f*^2^ = .02), *N* = 395 was required. Thus, our sample was large enough to detect very small partial correlation coefficients. The population of MTurkers has not been found to be representative of the U.S. population; for instance, MTurkers were reported to be more educated, more often unemployed, and more often Caucasian, as well as more frequently agnostic or atheist than in the general U.S. population, but much less often retired: 1.3% versus 21% [46]. This study was approved by the research ethics commission of the University of Basel and was conducted in accordance with the principles expressed in the Declaration of Helsinki. The data for this study are available on the Open Science Framework platform (https://osf.io/5bz2r/).

### Procedure

Participants completed an online questionnaire in January 2020. An informed consent form was displayed to participants as part of the task description on Mechanical Turk and on the first page of the online questionnaire. On the informed consent form, participants were informed of the general aim of the study, that they would fill in different questionnaires related to that aim, and of their right to withdraw from the study with no consequences other than forfeiting their compensation. Participants were compensated at the U.S. federal hourly minimal wage of USD 7.25 (USD 3.05 for an estimated 25 min of their time). We included attention checks as an additional step in screening inadequate participation. This has been shown to lead to higher quality of data in MTurkers than in subject pool participants [47]. The first attention check was presented as an independent question and is provided in the S1 Appendix. The second and third attention checks were additional items embedded in the TQ (“Select moderately here”) and the Fear of Spiders Questionnaire (FSQ; “Currently, I pay attention. Select ‘five’ to show attention to spider questionnaires”).

### Measures

All measures were self-reported and had adequate to high reliability (all Cronbach’s alphas > .71). The predictor variables used in this study were behavioral inhibition; the Big Five personality dimensions: neuroticism, conscientiousness, extraversion, agreeableness, and openness to experience; anxiety sensitivity; trait anxiety; disgust sensitivity; and disgust propensity.

#### Behavioral inhibition

To measure behavioral inhibition, we used the 16-item Adult Measure of Behavioral Inhibition (AMBI [34]). Items are scored on a 3-point Likert scale (0 = *no/hardly ever*, 1 = *some of the time*, and 2 = *yes/most of the time*). We used the AMBI total score, which showed good reliability (reported alpha = .87), although subscores could also be computed: fearful inhibition (example item: “Do you tend to observe strangers from a distance first, before being able to mix in?”), non-approach (e.g., “Do you tend to introduce yourself to new people?”, reversed), low sociability (e.g., “Do you tend to choose solitary leisure activities over spending time with close friends?”), and risk avoidance (e.g., “If physically able, would you enjoy adventure holidays with some element of risk?”). Reported alphas ranged from .52 to .86. The AMBI total score has a strong correlation with the total score of the Retrospective Measure of Behavioral Inhibition, a retrospective measure of childhood temperament (*r* = .73; [34]; for related work on specific phobias using the latter scale, see [36]). In our sample, Cronbach’s alpha was .82.

#### Big five personality traits

The HEXACO–60 [48] is a 60-item instrument measuring the Big Five personality traits and honesty–humility (which we did not analyze in this study). It is composed of the following subscales: Emotionality (= neuroticism, example item: “I would feel afraid if I had to travel in bad weather conditions”), extraversion (e.g., “The first thing that I always do in a new place is to make friends”), agreeableness (e.g., “My attitude toward people who have treated me badly is ‘forgive and forget’”), conscientiousness (e.g., “I plan ahead and organize things, to avoid scrambling at the last minute”), openness to Experience (e.g., “I’m interested in learning about the history and politics of other countries”), and honesty–humility (e.g., “I wouldn’t use flattery to get a raise or promotion at work, even if I thought it would succeed”). Each subscale has 10 items, with good reported internal reliabilities (all alphas > .72). The items are scored on a 5-point Likert scale (ranging from 1 = *strongly disagree* to 5 = *strongly agree*). The HEXACO has been used most frequently in personality psychology but has also been used in clinical psychology (more rarely) and other fields [49]. We note that much of the research relating personality to specific phobia used the Eysenck Personality Inventory [50] or the Eysenck Personality Questionnaire [51], with which one cannot assess the five-factor model of personality. In the present study, Cronbach’s alphas were .72 for neuroticism, .77 for extraversion, .73 for agreeableness, .79 for conscientiousness, and .76 for openness to experience.

#### Anxiety sensitivity

Anxiety sensitivity was assessed with the Anxiety Sensitivity Index–3 [52]. This 18-item instrument is composed of three subscales (six items each) with good reliability (all reported alphas > .78): physical concerns (“When my stomach is upset, I worry that I might be seriously ill”), cognitive concerns (“When my thoughts seem to speed up, I worry that I might be going crazy”), and social concerns (“It is important for me not to appear nervous”). Items are scored on a 5-point Likert scale (0 = *very little* to 4 = *very much*). We used the total sum score in our study, for which Cronbach’s alpha was .95.

#### Trait anxiety

Trait anxiety was assessed with the short form of the trait version of the State Trait Anxiety Inventory (STAI; six items [53]). Items (e.g., “I feel upset”) are scored on a 4-point Likert scale ranging from 1 = *not at all* to 4 = *very much*. The reported correlation of the short form with the full-length STAI is .9. In our study, Cronbach’s alpha was .82.

#### Disgust propensity and sensitivity

Disgust propensity and disgust sensitivity were measured using the Disgust Propensity and Sensitivity Scale–Revised ([32]), composed of 16 items, eight for each subscale (good reliability: both alphas > .86). The items are scored on a 5-point Likert scale (from 1 = *never* to 5 = *always*). Example items include “Disgusting things make my stomach turn” for disgust propensity and “When I feel disgusted, I worry that I might pass out” for disgust sensitivity. In our study, Cronbach’s alphas were .87 for disgust propensity and .89 for disgust severity.

The DVs used in this study were symptoms of trypophobia, spider phobia, and blood and injection phobia.

#### Symptoms of trypophobia

For the assessment of symptoms of trypophobia, our main DV, we used the TQ [16], composed of 17 items. The items (e.g., “Feel uncomfortable or uneasy” [when looking at trypophobic stimuli]) are scored on a Likert scale from 1 = *not at all* to 5 = *extremely*. The instrument has an excellent reported reliability (reported alpha = .96). In our study, Cronbach’s alpha was .98.

#### Symptoms of blood and injection phobia

Symptoms of blood and injection phobia were measured using the 17-item (response options: Yes/No) Blood-Injection Symptom Scale [54]. We used the total score, for which the reliability is good (reported alpha = .86), although three subscales can be computed with questionable reliability (alphas ranging from .56 to .72): the faintness subscale (example item: “Were you dizzy or lightheaded?” [when confronted with situations involving blood or injections]), the anxiety subscale (e.g., “Were you anxious?”), and the tension subscale (“Were you particularly irritable?”). In our study, Cronbach’s alpha was .90.

#### Symptoms of spider phobia

Spider phobia was assessed using the 18-item FSQ [55]. Items are rated on an 8-point Likert scale (0 = *strongly disagree* to 7 = *strongly agree*). An example item is “If I came across a spider now, I would get help from someone else to remove it”. The questionnaire has good reliability (alpha = .92). In our study, Cronbach’s alpha was .98.

#### Sociodemographic variables

We recorded age, gender, occupation, and educational attainment) as sociodemographic variables. Table 1 presents this information for our sample.

**Table 1:** Sociodemographic variables.

Variable	Frequency	Percent
Gender		
Men	603	53.17
Women	531	46.83
Employment status		
Working full-time	922	81.31
Working part-time	105	9.26
Unemployed	47	4.14
On parental leave	5	0.44
Retired	23	2.03
Other	32	2.82
Education		
Primary school	1	0.09
High school	94	8.29
Some college/university	196	17.28
Graduated from college/university	596	52.56
Master’s/postgraduate	233	20.55
Doctoral level	12	1.06
Other	2	0.18

*Note*. *N* = 1,134. Participants were on average 38.26 years old (*SD* = 10.59).

### Data analysis

We performed *t* tests of mean differences in the study variables between women and men and zero-order correlation tests of these variables with age in R. As we expected gender and age to be related to the study variables, we used partial correlation coefficients when computing associations between study variables (in SPSS), controlling for gender and age. For all analyses, the significance threshold was *p* < .05.

## Results

Table 2 presents the descriptive statistics and *t* tests for mean differences between women and men for the scales used in this study as well as zero-order correlations of these variables with age. It can be seen that men scored lower on the majority of the scales but higher on extraversion. Age is negatively related to most variables but positively related to extraversion, conscientiousness, and openness to experience. Agreeability is associated with neither gender nor age. The partial correlation coefficients between our study variables are presented in Table 3. Those between trypophobia, spider phobia, and blood and injection phobia and all other constructs thereby refer to the hypotheses as stated above (shaded area in Table 3).

**Table 2:** Descriptive statistics (*N* = 1,134).

Variable	Range	*M*	*SD*	*Median*	*t*_Gender_	*r*_Age_
AMBI	0–32	18.86	5.84	18	−3.47 *	−0.06 *
N^#^	1–5	3.17	0.63	3.2	−10.52 *	−0.06 *
E^#^	1–5	3.10	0.71	3.2	3.67 *	0.11 *
A^#^	1–5	3.30	0.64	3.2	0.11	0.04
C^#^	1–5	3.54	0.7	3.5	−3.80 *	0.18 *
O^#^	1–5	3.46	0.69	3.3	−0.34	0.08 *
ASI	0–72	29.69	18.01	31	−0.27	−0.17 *
STAI	0–24	5.94	4.09	6	−2.79 *	−0.15 *
D. PRO	8–40	23.46	6.59	24	−4.49 *	−0.03
D. SEN	8–40	21.20	7.72	21	−1.20	−0.14 *
TQ	17–85	37.95	19.71	33	0.38	−0.18 *
BISS	0–17	4.94	4.77	4	−2.40 *	−0.08 *
FSQ	0–126	49.64	39.45	53	−2.53 *	−0.16 *

*Note*. AMBI: Adult Measure of Behavioral Inhibition; N: Neuroticism (emotionality); E: Extraversion; A: Agreeableness; C: Conscientiousness; O: Openness to experience; ASI: Anxiety Sensitivity Index; STAI: State Trait Anxiety Inventory (trait scale); D. PRO: Disgust propensity; D. SEN: Disgust sensitivity; TQ: Trypophobia Questionnaire; BISS: Blood and Injection Symptom Scale; FSQ: Fear of Spiders Questionnaire.

^#^:Average scores computed; for all other scales we used sum scores.

*t*_Gender_: *t*-test of mean differences between the sexes with 1,132 degrees of freedom (a positive value represents a higher mean for men). *r*_Age:_ Zero-order correlation of age with the study variables.

*: *p* < .05.

**Table 3:** Partial correlations among the study variables (*N* = 1,134).

Variable	1	2	3	4	5	6	7	8	9	10	11	12	13
1. AMBI	–												
2. N	.28	–											
3. E	−.71	−.31	–										
4. A	−.10	−.07	.23	–									
5. C	.22	−.06	.*02*	.26	–								
6. O	.*04*	−.*01*	.11	.26	.44	–							
7. ASI	.*02*	.34	−.09	−.22	−.52	−.31	–						
8. STAI	.24	.38	−.44	−.32	−.32	−.24	.48	–					
9. D. PRO	.10	.31	−.10	−.17	−.29	−.24	.68	.40	–				
10. D. SEN	.*02*	.33	−.07	−.18	−.47	−.34	.83	.49	0.81	–			
11. TQ	−.16	.13	.10	−.15	−.55	−.37	.72	.38	.60	.72	–		
12. BISS	.07	.20	−.10	−.15	−.18	−.13	.47	.37	.42	.49	.46	–	
13. FSQ	−.07	.22	.*01*	−.14	−.45	−.36	.69	.36	.60	.68	.72	.42	–

*Note*. Shaded areas refer to the hypotheses as stated in the introduction. Correlation coefficients higher than .058 are significant at *p* < .05. Correlations in italics are *not* significant. AMBI: Adult Measure of Behavioral Inhibition; N: Neuroticism (emotionality); E: Extraversion; A: Agreeableness; C: Conscientiousness; O: Openness to experience; ASI: Anxiety Sensitivity Index; STAI: State Trait Anxiety Inventory (trait scale); D. PRO: Disgust propensity; D. SEN: Disgust sensitivity; TQ: Trypophobia Questionnaire; BISS: Blood and Injection Symptom Scale; FSQ: Fear of Spiders Questionnaire.

### Associations with trypophobia

Behavioral inhibition was negatively related to trypophobia, contrary to H1A. Neuroticism (emotionality) was positively associated with trypophobia. This confirmed H2A. Conscientiousness was negatively linked to trypophobia, as predicted in H3A. Anxiety sensitivity was positively related to trypophobia (H4A). Trait anxiety was positively associated with trypophobia as well (H5A). Disgust propensity and disgust sensitivity were both positively related to trypophobia (H6A and H7A). Contrary to H8A, women (*M* = 37.71, *SD* = 19.71) did not score higher on trypophobia symptoms than men (*M* = 38.15, *SD* = 19.73), *t* = 0.375, *p* = .707, Cohen’s *d* = 0.02.

### Associations with spider phobia

Behavioral inhibition was negatively related to spider phobia symptoms, contrary to H1B. Neuroticism (in agreement with H2B), conscientiousness (negatively; H3B), anxiety sensitivity (H4B), trait anxiety (H5B), disgust sensitivity (H6B), and disgust propensity (H7B) were all related to spider phobia symptoms in the expected direction. As hypothesized (H8B), women (*M* = 52.79, *SD* = 39.54) scored higher on spider phobia than men (*M* = 46.87, *SD* = 39.21), *t* = -2.529, *p* = .012, Cohen’s *d* = 0.15.

### Associations with blood and injection phobia

Hypotheses H1C to H7C were all confirmed for blood and injection phobia, and again associations pointed in the directions we expected. Further, as expected (H8C), women (*M* = 5.31, *SD* = 4.93) presented more blood and injection phobia symptoms than men (*M* = 4.63, *SD* = 4.61), *t* = -2.399, *p* = .017, Cohen’s *d* = 0.14.

### Exploratory analyses

We analysed the associations between trypophobia, spider phobia, and blood and injection phobia and extraversion, agreeableness, and openness to experience exploratively. Results showed that agreeableness and openness to experience were negatively related to symptoms of trypophobia, spider phobia, and blood and injection phobia (see Table 3). Extraversion was positively associated with symptoms of trypophobia but negatively with symptoms of blood and injection phobia.

## Discussion and conclusion

The aims of the current study were (a) to draw the contours of the nomological network of trypophobia by assessing the associations of a range of constructs related to the three anxiety disorder variables, and specific phobias in particular, with trypophobia; (b) to examine similarities and difference in such associations in comparison with those found with spider phobia and blood and injection phobia; and (c) to investigate the main effect of gender on trypophobia. We also wanted to replicate the association of gender with spider phobia and blood and injection phobia.

We hypothesized that conscientiousness would be negatively related to these three variables (H3A to H3C), whereas the following constructs would be positively related to them: behavioral inhibition (H1A to H1C), neuroticism (H2A to H2C), anxiety sensitivity (H4A to H4C), trait anxiety (H5A to H5C), disgust sensitivity (H6A to H6C), and disgust propensity (H7A to H7C). Finally, we hypothesized that women would present with more symptoms of trypophobia, spider phobia, and blood and injection phobia (H8A to H8C).

### Similarities in the nomological networks

**Fig 1:**
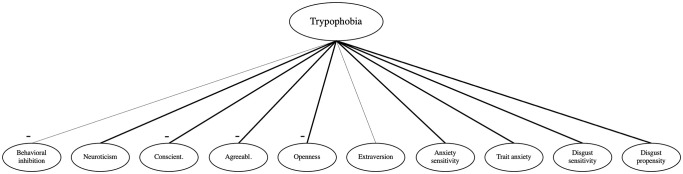
Nomological net of trypophobia. Thicker lines represent associations in common with spider phobia and blood and injection phobia.

A large majority of these hypotheses were supported in this study, as summarized in Table 4. We thus find the nomological network of trypophobia to be in appearance very similar to that of spider phobia and blood and injection phobia for the studied associations. Fig 1 presents a graphic summary of our results focusing on trypophobia symptoms, our main DV.

**Table 4:** Summary of the hypothesis tests.

Hypothesis	Variable	Trypophobia	Spider phobia	Blood and injection phobia
H1A–C	Behavioral inhibition (+)			X
H2A–C	Neuroticism (+)	X	X	X
H3A–C	Conscientiousness (−)	X	X	X
H4A–C	Anxiety sensitivity (+)	X	X	X
H5A–C	Trait anxiety (+)	X	X	X
H6A–C	Disgust sensitivity (+)	X	X	X
H7A–C	Disgust propensity (+)	X	X	X
H8A–C	Gender (women +)		X	X

*Note*. Rows are the independent variables and columns the dependent variables. (+): Positive relationship hypothesized; (−): Negative relationship hypothesized. X: Hypothesis is supported.

Indeed, we found higher symptoms of trypophobia, spider phobia, and blood and injection phobia in individuals who had high negative affectivity (neuroticism), were less conscientious, more worried about feeling anxious (anxiety sensitivity), and more anxious in general (trait anxiety), as well as individuals who experienced disgust frequently (disgust propensity) and were apprehensive about this emotion (disgust sensitivity). Regarding the exploratory analyses of the associations of other dimensions of personality with symptoms of trypophobia, spider phobia, and blood and injection phobia, we found that both agreeableness and openness to experience were negatively associated with all the DVs.

### Differences in the nomological networks

Despite these similarities, several differences could be observed. The association of withdrawal from unfamiliar situations (behavioral inhibition) was different for different target variables: It was positively associated with blood and injection phobia but negatively associated with trypophobia and spider phobia. Considering the exploratory analyses of the associations of other dimensions of personality with our DVs, it is worth noting that extraversion was positively associated with trypophobia, whereas it was negatively associated with blood and injection phobia and non-significantly associated with spider phobia. Another difference between trypophobia and the other target variables was that gender was not associated with trypophobia, whereas it was associated with spider phobia and blood and injection phobia (higher scores for women). These differences suggest the nomological network of trypophobia to be in part different from those of spider phobia and blood and injection phobia.

Examining the effect sizes in the tests of hypotheses pointed to further differences in the nomological networks. We relied on Cohen’s [56] suggested thresholds for small, moderate, and large effect sizes (for H1A to H7C, partial *r* = .1: small effect; partial *r* = .3: moderate effect, partial *r* = .5: large effect; for H8A to H8C, Cohen’s *d* = 0.2: small; *d* = .5: moderate, *d* = 0.8: large). Effect sizes appeared to be globally lower with regard to the associations of the independent variables with blood and injection phobia symptoms (H1C, H2C, H3C, H4C, H5C, H6C, H7C; average absolute value of partial *r*s = .26; on average, small effect sizes) compared with trypophobia symptoms (H1A, H2A, H3A, H4A, H5A, H6A, H7A; average absolute value of partial *r*s = .39; moderate) and spider phobia (H1B, H2B, H3B, H4B, H5B, H6B, H7B; average absolute value of partial *r*s = .36; moderate). Further examining the individual partial correlations (Table 3), one can see that the partial correlations testing hypotheses related to blood and injection phobia symptoms never crossed Cohen’s threshold for large effect sizes, whereas this occurred for most of those related to trypophobia symptoms (H3A, H5A, H6A, H7A), and three of those related to spider phobia (H4B, H6B, H7B). The most important difference in effect sizes between the associations with the DVs relates to conscientiousness: the effect size was large for trypophobia symptoms, moderate for spider phobia symptoms, and small for blood and injection phobia symptoms.

### Strengths and limitations

One strength of our study was the well-balanced number of women and men participants, as this allowed the studied associations not to be driven by relationships found in one gender in particular. A second strength is that the sample size for this study, considerably higher than for most studies interested in trypophobia, afforded the detection of effects of different magnitudes (ranging from small to large). Further, relying upon partial correlation tests allowed us to obtain estimates of association that were concise, comparable across instruments, and easily interpretable. A limitation of this study is that our sample is not representative of the general population, and we cannot guarantee that our findings could extend to other populations. This is because inferential statistics do not allow generalizing beyond the population from which the sample used for the analyses is drawn. Yet, it should be noted that findings similar to ours, in the relationships of specific phobia and the independent variables formerly assessed in the literature, are common (e.g., [32,33,36–38,41,44,45]). This study did not aim at investigating temporal relationships between independent and dependent variables and hence relied upon cross-sectional data. This is not per se a limitation, but further studies could explore such relationships.

In conclusion, the results of our study draw a clearer picture of the nomological net of trypophobia (depicted in Fig 1). That in the present study the nomological network of trypophobia includes many of the constructs related to specific phobias in past research hints at the possibility that trypophobia is also a specific phobia. But because we observed not only similarities but also differences between trypophobia and spider phobia as well as blood and injection phobia in terms of associations with the constructs of interest and their effect sizes, further studies are needed to clarify similarities and dissimilarities between trypophobia and specific phobia. Also, structural equation models could be used to test specific hypotheses about similarities and dissimilarities between the three target variables with respect to their associations with all other constructs assessed.

## Supporting information

S1 Appendix (DOCX)
