# Supplementary material for: An exploration of the nomological network of trypophobia
Source: PLoS One. 2021 Sep 14;16(9):e0257409. doi: 10.1371/journal.pone.0257409 (PMC8439462; doi:10.1371/journal.pone.0257409)
Supplement: S1 Appendix — (DOCX) [file pone.0257409.s001.docx]

**Appendix**

**Independent attention check (adapted from [1])**

Most modern theories of emotions recognize that affects do not take place in a vacuum. Individual experiences and knowledge along with situational variables can greatly impact the affective states. In order to facilitate our research on anxiety and emotions we are interested in knowing certain factors about you. Specifically, we are interested in whether you actually take the time to read the directions; if not, then our measures that rely on accurately reading the instructions and answering accordingly will be ineffective. So, in order to demonstrate that you have read the instructions, please ignore the emotional items below. Instead, select the box marked Other and type “I read the instructions” (no quotes) in the text box, then click continue.

Which of these emotions are you feeling at the moment? (click on all that apply)

The listed emotions were pride, elation, joy, satisfaction, relief, hope, interest, surprise, sadness, fear, shame, guilt, envy, disgust, contempt, anger, and other, below which an input text field was provided.

**References**

[1] Berinsky AJ, Margolis MF, Sances MW. Can we turn shirkers into workers? J Exp Soc Psychol. 2016; 66: 20-28.
